# Development and Validation of the Schedule for the Assessment of Insight in Anxiety Disorders (SAI-A)

**DOI:** 10.1192/j.eurpsy.2026.10788

**Published:** 2026-07-31

**Authors:** A. Halaj, A. S. David, J. D. Huppert, K. George

**Affiliations:** 1 University of Haifa, Haifa, Israel; 2 University College London, London, United Kingdom; 3 The Hebrew University of Jerusalem, Jerusalem, Israel; 4 University of Athens, Athens, Greece

## Abstract

**Introduction:**

Insight, the awareness and understanding of one’s mental disorder plays a significant role in characterizing various psychological disorders. While much research on insight has focused on psychosis, there is a growing interest in understanding insight or illness awareness in anxiety, often assessed by instruments designed for psychosis. There is a growing interest in understanding insight or illness awareness in anxiety, however, most assessment instruments were designed for psychosis. The unique features of anxiety highlight the need for tailored measures to accurately evaluate insight.

**Objectives:**

The aim of this study is to develop and validate the Schedule for the Assessment of Insight in Anxiety (SAI-A), a clinician-rated scale for assessing insight in anxiety disorders.

**Methods:**

We interviewed 46 participants diagnosed with anxiety disorders, conducted SAI-A interviews, and administered self-report measures. Using correlation and principal component analysis, we identified and assessed scale components, ensuring their reliability and consistency.

**Results:**

The SAI-A demonstrated acceptable psychometric properties, including convergent validity with an established self-report measure (*r* = -0.39, *p* = 0.008) and internal consistency (Cronbach’s alpha = 0.70). It showed moderate to strong agreement, interrater reliability (weighted kappa = 0.53, ICC = 0.67), and test-retest reliability (ICC = 0.65). Two distinct insight components emerged: awareness of disorder and need for treatment. Higher overall SAI-A scores correlated with symptom severity and impairment (*r* = 0.56, *r* = 0.51, *p* < 0.001, respectively) and medication usage.

**Conclusions:**

The SAI-A is a valid and reliable assessment tool, providing a comprehensive framework for understanding and addressing insight in the context of anxiety disorders. This new assessment tool bridges existing research gaps and offers a tailored approach to assessing insight, with the goal to improve clinical understanding for those facing these challenges.

**Disclosure of Interest:**

None Declared

